# Effects of Chewing Gum on Plaque Index: A 3-Dimensional Colorimetric Analysis

**DOI:** 10.3390/dj13100474

**Published:** 2025-10-17

**Authors:** Luca Levrini, Piero Antonio Zecca, Virginia Bellora, Alessandro Deppieri, Stefano Saran, Nicola Giannotta, Andrea Carganico

**Affiliations:** 1 Department of Human Science and Innovation for the Territory, University of Insubria, 21100 Varese, Italy; luca.levrini@uninsubria.it; 2 Department of Medicine and Innovative Technologies, University of Insubria, 21100 Varese, Italy; pieroantonio.zecca@uninsubria.it; 3 School of Medicine, University of Insubria, 21100 Varese, Italy; vbellora2@studenti.uninsubria.it (V.B.); adeppieri@studenti.uninsubria.it (A.D.); ssaran@studenti.uninsubria.it (S.S.); ngiannotta@studenti.uninsubria.it (N.G.); 4 Department of Biotechnology and Life Sciences, University of Insubria, 21100 Varese, Italy

**Keywords:** dental plaque, chewing gum, biofilms, colorimetry, oral hygiene, plaque index

## Abstract

**Background:** This study aimed to evaluate the effectiveness of xylitol- and fluoride-containing chewing gum in reducing dental plaque using a novel 3D colorimetric analysis, and to compare results with the Plaque Control Record (PCR). **Methods:** An acute intervention study was conducted on 34 healthy adults (18–45 years). A plaque-disclosing solution was applied, and intraoral scans were taken before and after 15 min of gum mastication. Plaque was quantified with PCR and the Placca Read software, which analyzes colorimetric patterns of scanned images. Statistical analyses (Shapiro–Wilk test, paired *t*-test) were performed with Jamovi Software version 1.6.14. **Results:** A significant reduction in plaque scores was observed after chewing gum (*p* < 0.05). Mean reduction reached −14.8% in the experimental group versus −3.9% in controls, where natural saliva flow and pigment washout may explain the modest decline. The 3D analysis provided precise measurements across all dental surfaces and showed strong correlation with PCR, supporting its validity. **Conclusions:** These findings indicate that functional chewing gum can significantly reduce plaque accumulation even after a single use, and that 3D colorimetric analysis offers a reliable, comprehensive alternative to conventional indices.

## 1. Introduction

Dental plaque remains a significant focus of oral health research due to its critical role in the development of caries and periodontal disease [1,2]. This biofilm, comprising diverse microbial communities, forms on tooth surfaces and can contribute to gingival inflammation and tooth decay if not adequately managed. Conventional mechanical cleaning methods, including brushing and flossing, are essential in controlling plaque; however, they are often insufficient in populations with limited access to dental care or in individuals with specific conditions, such as orthodontic appliance users [3]. In such cases, complementary strategies, such as the use of functional chewing gums, have emerged as promising adjuncts to oral hygiene regimens [4].

Functional chewing gums, particularly those containing xylitol or other active antimicrobial agents, have been shown to offer significant oral health benefits [1,2,3]. Sugar-free chewing gum has been found to enhance saliva flow, aiding in the neutralization of acids and the mechanical clearance of food particles and bacteria [5,6,7,8]. Systematic reviews have consistently demonstrated reductions in plaque accumulation with xylitol gum compared to control gums containing sorbitol or no active ingredients [5,9].

Emerging evidence also suggests that chewing gums with additional antimicrobial properties, such as those containing essential oils or bioactive compounds, may further enhance oral health outcomes [10]. For instance, gums enriched with essential oils like peppermint and cinnamon have shown the potential to reduce plaque and gingival inflammation. This is particularly relevant for orthodontic patients, who face increased plaque retention due to the presence of brackets and wires, which create niches for bacterial colonization. A recent randomized controlled trial highlighted the efficacy of antimicrobial chewing gums in reducing approximal plaque and gingival indices over a 30-day period [10,11].

In addition to antimicrobial and plaque-reducing effects, functional gums can positively influence the oral microbiota. Studies utilizing advanced sequencing technologies, such as 16S rDNA analysis, have shown that xylitol gum use can decrease the relative abundance of pathogenic bacterial species, including Firmicutes, Bacteroidetes, and Actinobacteria, while promoting the growth of beneficial taxa like Fusobacteria [3,7,12]. These findings underscore the potential of functional gums to shift the microbial ecosystem towards a state of symbiosis, thereby reducing the risk of caries and periodontal diseases [7,12].

Despite these promising results, the integration of chewing gums as a routine preventive measure in oral health care requires further validation. Many studies to date have relied on traditional plaque indices, which may not capture the full scope of plaque distribution and composition. However, traditional plaque indices rely on subjective scoring and often underestimate plaque accumulation on occlusal and interproximal surfaces, underscoring the need for more objective and comprehensive methods such as 3D colorimetric analysis. Novel three-dimensional (3D) colorimetric methods have emerged as innovative tools to provide detailed quantitative assessments of plaque distribution on all tooth surfaces, including occlusal and interproximal areas that are typically underrepresented in conventional analyses [13]. This approach offers a more comprehensive evaluation of the efficacy of chewing gums in plaque reduction [13].

This study builds on previous research by investigating the effects of a functional chewing gum on dental plaque using a 3D colorimetric method. By comparing outcomes with traditional plaque indices, it aims to validate the utility of this innovative technology and provide a deeper understanding of the role of functional gums in enhancing oral hygiene. Additionally, this research seeks to address gaps in current knowledge by exploring the interaction between functional gum use and dental plaque.

In conclusion, the integration of functional chewing gums into oral hygiene routines holds significant promise as a complementary strategy for managing dental plaque. This study endeavors to advance our understanding of their efficacy, utilizing state-of-the-art assessment methods to inform clinical and public health recommendations.

## 2. Materials and Methods

This was an acute intervention study which involved healthy subjects (adults 18–45 years). A sample size of 34 subjects is required to detect an effect size of 1, with statistical power set at 0.8 (significance α = 0.05). Sample size was calculated using Jamovi Software (version 1.6.14, Jamovi Project, Sydney, Australia) based on a pilot study.

All recruited subjects fulfilled the following criteria for participation in this study: nonsmokers, absence of extensive dental restorations or adhesive fixed partial dentures, good general and initial periodontal health, and obligatory lack of antibiotic therapy 1 month before the beginning of the study and during the study, and no usage of anti-plaque and oral antiseptic solutions during the entire investigation. Exclusion criteria were: systemic conditions, xerostomia and presence of oral disorders. In order to have a homogeneous sample, subjects with Class I skeletal relationship, normo-divergent, Class I molar relationship, correct overjet, correct overbite and with minimal irregularity in the range of crowding according to Little’s Index were selected (calculated as the sum of the linear contact point displacements of the six mandibular anterior teeth).

All the subjects were instructed to adopt the following domiciliary oral hygiene regime 1 week before the study: to use a soft toothbrush with a rolling-action technique with a fluoride toothpaste 2 times a day, and dental floss 1 time a day. The same toothbrush (Mentadent Professional, Unilever, London, UK) and toothpaste (Mentadent Professional, Protect Plus, Unilever, London, UK) were provided to the subjects.

The study was carried out in accordance with the principles of the Declaration of Helsinki and in compliance with Good Clinical Practice. Before taking part in the study, each patient was required to sign an informed consent form to which was attached a detailed description of the study protocol (C.E. Università degli Studi dell’Insubria. n. 0111335 23 December 2022).

The gum used in this study is a common chewing gum (Daygum Microtech, Perfetti Van Melle, Milan, Italy) a sugar-free chewing gum formulated with polyol sweeteners (sorbitol, maltitol, mannitol, xylitol) and fluoride. It is marketed in the form of coated dragees available at food stores, bars, supermarkets in Italy, while the plaque disclosing solution and dental scan are standard tools within dental practice. As a matter of fact, the procedures and protocols of this study reproduce ordinary daily life activities.

Subjects were asked to suspend oral hygiene procedures for 12 h before the examination.

To obtain a control value a plaque disclosing solution (Miradent, Duisburg, Germany) was applied on teeth surfaces and an intraoral scansion was recorded (control T0) using an iTero scanner (Align Technology Inc., San Jose, CA, USA). Participants were asked to wait 15 min, and a second intraoral scansion was recorded (control T1).

After the complete dissolution of the plaque disclosing solution, a second application of the plaque disclosing solution was then delivered on teeth surfaces and another intraoral scansion was recorded using an iTero scanner (experimental T0). Participants were asked to chew a chewing gum for 15 min; afterwards the plaque disclosing solution was applied again, and another intraoral scansion was recorded (experimental T2).

The images from the intraoral scansion were exported, segmented to remove the gingival tissue, and uploaded in the “Placca Read Software” (Insubria University, Varese, Italy). This software allows the measurement of the number of pixels for each dental scansion attributable to the plaque disclosing solution by analyzing the colorimetric pattern of the images. The Placca Read software identifies disclosed plaque by applying standardized color thresholds in the red spectrum corresponding to the disclosing solution. Prior to analysis, the software was calibrated using reference images of known plaque coverage to ensure reproducibility. This process ensured that the software could provide objective, quantitative measurements of plaque distribution across all dental surfaces. A plaque score is then provided for each subject, calculating for each image the number of pixels matching the plaque disclosing solution color. In particular, the software allows the evaluation of both the vestibular, the anterior and the occlusal surfaces of the teeth, which is typically not considered in the most used plaque indexes.

This innovative plaque scores were therefore compared before and after 15 min, with and without chewing gum.

**Figure 1:**
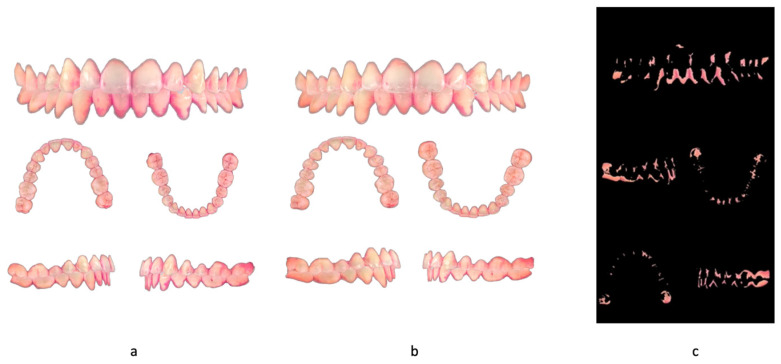
Images of the intraoral scansion. (**a**) before use of chewing gum. (**b**) after use of chewing gum. (**c**) difference before and after use of chewing gum.

Therefore, to validate the method the procedure was repeated a second time on 5 participants of the experimental group by a different operator, to assess the intraclass correlation coefficient (ICC). In addition, results were compared to the traditional plaque control record (PCR) evaluated directly on the same subjects measured with the dental scan (Figure 1). The PCR assessment was performed by a blinded examiner to minimize bias. Statistical analysis was performed using Jamovi Software. Shapiro–Wilk test was used to assess the normal distribution of the variables. Paired *t*-test was run to report any statistically significant change between the treatment and the control regarding the plaque score.

## 3. Results

The experimental protocol was completed without any unforeseen issues. The results obtained during the study are presented below.

**Figure 2:**
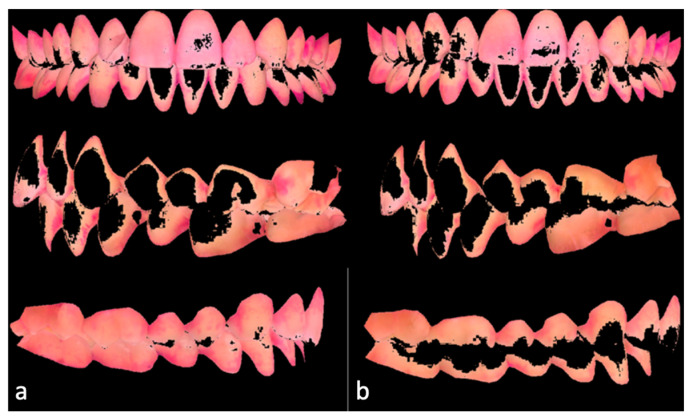
Placca Read software in one subject showing the pixel analysis: the pink color represents surfaces covered by plaque before (**a**) and after 15 min of chewing gum (**b**).

Table 1 shows the mean data collected using the Placca Read software for all the participants. For each quadrant, the first value corresponds to the initial digital scan, the second to the control scan taken 15 min after the first, and the last value to the final scan taken after 15 min of chewing gum (Figure 2). Statistical analysis of the data revealed that all variables examined in the study were statistically significant and consistent with the initial expectations. The Shapiro–Wilk test confirmed the normal distribution of the data, allowing for inferential analysis.

In the experimental results, participants who chewed Daygum Microtech gum for 15 min showed a significant reduction in plaque scores even after a single consumption. The three-dimensional colorimetric analysis conducted with Placca Read software allowed for precise measurement of the pixel count associated with the plaque-detecting solution. Results indicated a significant decrease in these pixels across all surfaces compared to control values, highlighting an effective reduction in plaque accumulation following gum use. The lower absolute reduction observed in the mandibular arch can be explained by the reduced baseline plaque values in this region, which were likely influenced by a more pronounced washout effect due to saliva pooling and tongue movements that accelerate the dilution and clearance of the disclosing agent.

In the control, participants who did not use the chewing gum also showed significant variations in plaque levels during the 15 min observation period. The second intraoral scan revealed a reduction in plaque-indicating pixels, suggesting that the simple passage of time contributed to a decrease in plaque accumulation or to some washout in the disclosing pigment. In fact, disclosing agents may be subject to partial dilution or removal by salivary flow and mechanical action in the oral cavity, which can reduce the apparent staining over time [14].

The colorimetric analysis results were compared to those obtained using the traditional plaque index assessment method, showing a strong correlation between the two approaches. This confirms the validity of the three-dimensional computerized approach for dental plaque analysis and suggests that it provides a reliable and detailed measure, including dental surfaces often overlooked in conventional analyses. The paired-samples *t*-test revealed a statistically significant difference (*p* < 0.05) between the changes observed in the experimental procedure and control, demonstrating the efficacy of chewing gum in reducing dental plaque. Regarding the validation of the procedure, the ICC showed a high degree of reliability (ICC > 0.97, 95% CI: 0.94–0.99). The comparison of the results with the PCR showed likewise a significant reduction after the use of the chewing gum: while a reduction of 4.1% in the PCR was observed in the control, a reduction of 15.2% was observed using the chewing gum.

## 4. Discussion

The findings of this study contribute significantly to the growing body of evidence supporting the use of functional chewing gums as effective adjuncts to conventional oral hygiene practices. Using a novel three-dimensional (3D) colorimetric method, our results demonstrated a significant reduction in dental plaque accumulation following the use of chewing gum. The plaque-reducing effect observed can be explained by multiple mechanisms: the mechanical action of chewing, which dislodges loosely adherent biofilm; the stimulation of salivary flow, which increases clearance of food debris and neutralizes acids; and the activity of the gum’s functional ingredients, such as xylitol and fluoride, which inhibit bacterial metabolism and promote remineralization. The combined contribution of these factors likely accounts for the consistent reductions measured in our study.

Our findings align with prior studies documenting the plaque-reducing effects of chewing gums in randomized controlled trials [1,3]. In agreement with existing literature, we observed a marked decrease in plaque scores, underscoring the efficacy of chewing gums as a biofilm disruptor. The ability to inhibit bacterial adherence and metabolism, particularly in cariogenic species like Streptococcus mutans, was a key mechanism suggested in earlier reviews [3,5,15]. The systematic nature of these prior investigations lends additional credibility to our conclusions, demonstrating the replicability of outcomes across diverse population groups and methodologies.

Clinical trials involving antimicrobial chewing gums enriched with essential oils have shown comparable efficacy in reducing plaque and gingivitis over extended intervention periods [2,7]. Although our study did not include essential oils, the outcomes reinforce the concept that functional chewing gums can substantially benefit oral health, particularly in reducing biofilm accumulation. Interestingly, while prior research highlighted improvements in gingival health, our focus remained primarily on plaque indices. This difference in focus points to the complementary nature of these studies and suggests the potential for combining xylitol and essential oils to maximize therapeutic effects [7,16].

The results of this study suggest that chewing gum use is associated with a measurable reduction in plaque accumulation, supporting the hypothesis that it may represent an effective plaque control strategy. Furthermore, the innovative 3D colorimetric technique proved effective and promising for providing a detailed and quantitative assessment of plaque, paving the way for further clinical and research applications.

Furthermore, our study’s findings align with microbiome analyses [3,10]. The 3D colorimetric analysis used in our study provides complementary evidence, focusing on plaque volume and distribution rather than microbial composition. Together, these approaches offer a holistic understanding of xylitol’s effects on oral biofilms. This integration of microbiological and physical data underscores the multi-dimensional benefits of functional gum in managing oral health [10,12,17].

While most prior studies report positive outcomes with xylitol gum, some inconsistencies exist. Certain investigations observed varying results depending on the dose and duration of xylitol exposure [5,6,18]. In our study, participants chewed gum for a defined period, which may explain the consistent reductions observed. However, earlier findings noted that prolonged or high-dose xylitol use could lead to diminishing returns, suggesting a need for optimization of usage protocols [6]. This insight highlights the necessity of carefully calibrating xylitol dosage in clinical recommendations to ensure sustained efficacy.

Another point of divergence relates to the methodology. Traditional plaque indices, often relying on subjective scoring systems, may not capture subtle changes in plaque distribution. Our use of a 3D colorimetric method addressed this limitation, providing high-resolution, quantitative data on plaque accumulation across all tooth surfaces, including areas typically overlooked in conventional analyses [13,19]. This methodological advancement emphasizes the importance of precision in evaluating oral hygiene interventions and sets a new standard for future research.

The clinical relevance of these findings is underscored by the growing interest in adjunctive therapies for plaque control, particularly in populations with compromised oral hygiene, such as orthodontic patients or those with limited manual dexterity [2,8,20]. The observed reduction in plaque accumulation suggests that functional gums could serve as an accessible, low-cost intervention to supplement routine brushing and flossing. Moreover, the potential to combine xylitol with other bioactive agents, such as essential oils or enzymes, could further enhance therapeutic outcomes, making this approach adaptable to a wide range of clinical needs.

Despite the strengths of our study, certain limitations should be acknowledged. The relatively short duration of the intervention limits our ability to assess long-term effects. A limitation of this study is that only one chewing gum formulation and a fixed chewing time were tested, thus the potential effects of different compositions or chewing durations could not be evaluated. Additionally, while the 3D colorimetric method provides detailed quantitative data, it does not offer insights into microbial composition or metabolic activity. Future studies integrating microbiome analyses with advanced imaging techniques could provide a more comprehensive understanding of functional gums’ impact on oral ecosystems [10,18]. Such interdisciplinary approaches would enhance the robustness of findings and pave the way for innovative therapeutic strategies.

Another limitation is the sample size, which, while adequate for detecting significant differences in plaque scores, may not capture variations across diverse populations. Larger, multicenter trials are needed to validate these findings and explore the effects of chewing gums in different demographic groups, including children, elderly individuals, and those with systemic health conditions that affect oral health [5,6]. Addressing these gaps in research will be crucial for translating findings into actionable public health recommendations [21].

Our findings corroborate the growing evidence base for chewing gums as a viable adjunct in plaque control. The integration of advanced assessment technologies, such as 3D colorimetric methods, marks a significant step forward in oral health research, offering a more nuanced understanding of intervention efficacy. By bridging microbiological insights with physical measurements, this study lays the groundwork for a new era of precision in dental care research, with the potential to significantly improve patient outcomes across diverse populations [9,13,22,23,24].

Furthermore, the implications of these findings extend beyond individual oral health benefits. The incorporation of functional gums into public health strategies, particularly in resource-limited settings, could address widespread challenges in oral hygiene maintenance. The scalability and cost-effectiveness of such interventions make them promising candidates for broader implementation in community health programs [2,8]. Future research should investigate the socio-economic impact of deploying functional gum on a large scale, particularly in underserved populations where traditional dental care services are inaccessible [11].

Additionally, the synergistic use of xylitol with emerging technologies, such as personalized oral care devices, presents exciting opportunities for innovation. For instance, integrating xylitol-based interventions with AI-driven oral health monitoring tools could revolutionize the way individuals manage their oral hygiene [25]. These advancements could empower patients with real-time feedback on their oral health status, fostering a more proactive approach to dental care.

The durability of xylitol’s effects also warrants further investigation. While our study confirmed its short-term efficacy, exploring its impact over extended periods remains a priority. Longitudinal studies examining the cumulative benefits of xylitol use, particularly in preventing the progression of periodontal diseases, could provide valuable insights [6]. Moreover, assessing the effects of xylitol on specific subpopulations, such as individuals with diabetes or immunocompromised conditions, would enhance the generalizability of findings.

In conclusion, our research underscores the transformative potential of functional chewing gums in contemporary oral health management. By leveraging innovative assessment methods and building on existing evidence, this study contributes to a deeper understanding of how simple, cost-effective interventions can drive meaningful improvements in dental hygiene practices. Continued exploration in this domain will undoubtedly pave the way for more inclusive, effective, and accessible oral care solutions for all.

## 5. Conclusions

In conclusion, this study confirms the potential of functional chewing gum as an accessible and cost-effective adjunct to daily oral hygiene routines. Beyond demonstrating its short-term effectiveness in reducing plaque, the findings emphasize the importance of innovative assessment tools. The three-dimensional colorimetric method proved to be a reliable and detailed approach, offering clear advantages over traditional indices by capturing plaque distribution across all dental surfaces, including those often underestimated by conventional scoring. This technology could be applied not only in clinical trials but also in routine practice, where a more objective and reproducible evaluation of plaque may improve patient monitoring and personalized preventive strategies. Future research should explore the long-term benefits of functional gums and expand the use of 3D analysis to more diverse populations and oral health conditions.

## Figures and Tables

**Table 1:** Data after suspension of oral hygiene procedures (baseline T0), after 15 min (control T1) and after 15 min use of chewing gum (experimental T2). *p* value is referring to the comparison of control and experimental. Values are expressed in pixels.

	Baseline T0	Control T1	Δ	Experimental T2	Δ	*p*-Value
anterior	20.619	16.679	−3.940	5.780	−14.838	*p* < 0.05
right	20.289	16.462	−3.827	5.475	−14.813	*p* < 0.05
left	19.757	14.814	−4.943	5.279	−14.478	*p* < 0.05
superior	17.435	7.273	−10.162	4.308	−13.126	*p* < 0.05
inferior	9.646	5.749	−3.897	2.784	−6.862	*p* < 0.05

## Data Availability

The raw data supporting the conclusions of this article will be made available by the authors on request.
